# ALS-Associated TDP-43 Induces Endoplasmic Reticulum Stress, Which Drives Cytoplasmic TDP-43 Accumulation and Stress Granule Formation

**DOI:** 10.1371/journal.pone.0081170

**Published:** 2013-11-29

**Authors:** Adam K. Walker, Kai Y. Soo, Vinod Sundaramoorthy, Sonam Parakh, Yi Ma, Manal A. Farg, Robyn H. Wallace, Peter J. Crouch, Bradley J. Turner, Malcolm K. Horne, Julie D. Atkin

**Affiliations:** 1 Department of Biochemistry, La Trobe Institute for Molecular Science, La Trobe University, Bundoora, Victoria, Australia; 2 Florey Institute of Neuroscience and Mental Health, The University of Melbourne, Parkville, Victoria, Australia; 3 Queensland Brain Institute and School of Chemistry and Molecular Biosciences, The University of Queensland, Brisbane, Queensland, Australia; 4 Department of Pathology, The University of Melbourne, Parkville, Victoria, Australia; 5 Florey Department of Neuroscience and Mental Health, Faculty of Medicine, Dentistry & Health Sciences, The University of Melbourne, Parkville, Victoria, Australia; 6 Saint Vincent's Hospital, Fitzroy, Victoria, Australia; Institute of Health Science, China

## Abstract

In amyotrophic lateral sclerosis (ALS) and frontotemporal lobar degeneration, TAR DNA binding protein 43 (TDP-43) accumulates in the cytoplasm of affected neurons and glia, where it associates with stress granules (SGs) and forms large inclusions. SGs form in response to cellular stress, including endoplasmic reticulum (ER) stress, which is induced in both familial and sporadic forms of ALS. Here we demonstrate that pharmacological induction of ER stress causes TDP-43 to accumulate in the cytoplasm, where TDP-43 also associates with SGs. Furthermore, treatment with salubrinal, an inhibitor of dephosphorylation of eukaryotic initiation factor 2-α, a key modulator of ER stress, potentiates ER stress-mediated SG formation. Inclusions of C-terminal fragment TDP-43, reminiscent of disease-pathology, form in close association with ER and Golgi compartments, further indicating the involvement of ER dysfunction in TDP-43-associated disease. Consistent with this notion, over-expression of ALS-linked mutant TDP-43, and to a lesser extent wildtype TDP-43, triggers several ER stress pathways in neuroblastoma cells. Similarly, we found an interaction between the ER chaperone protein disulphide isomerase and TDP-43 in transfected cell lysates and in the spinal cords of mutant A315T TDP-43 transgenic mice. This study provides evidence for ER stress as a pathogenic pathway in TDP-43-mediated disease.

## Introduction

TAR DNA-binding protein 43 (TDP-43) is a protein constituent of pathologic cytoplasmic and intranuclear inclusions in neurons and glia of patients with sporadic and familial forms of amyotrophic lateral sclerosis (ALS) and frontotemporal lobar degeneration (FTLD) [1], [2]. While predominantly a nuclear protein, a proportion of TDP-43 is cytoplasmic, even under normal conditions [3], [4], [5]. When nuclear localisation sequences of TDP-43 are genetically ablated, the protein accumulates in the cytoplasm and forms inclusions that are similar to those seen in disease [4], [6]. Recently, it was shown that under cellular stress, TDP-43 accumulates in the cytoplasm and forms cytoplasmic stress granules (SGs) [7], [8], [9]. The sub-cellular location of the inclusions and the effects of TDP-43 inclusions on cellular physiology are not well known. Also, the relationship between SGs and inclusions remains controversial, although SGs could represent a precursor to TDP-43 inclusions [9], [10].

SGs form rapidly in response to a variety of cellular insults and lead to translational repression of incorporated mRNAs [11]. SG assembly is usually initiated by the phosphorylation of eukaryotic initiation factor 2 alpha (eIF2α), which inhibits formation of the ternary complex (eIF2/GTP/tRNA^Met^) required to initiate protein translation [12], [13]. Although the specific stressors which direct TDP-43 to SGs *in vivo* remain unclear, conditions including endoplasmic reticulum (ER) stress, heat shock, oxidative stress, osmotic stress, and serum deprivation can all cause TDP-43-positive SG formation in certain cell types in cell culture systems [14]. Interestingly, different cell types display different levels of recruitment of TDP-43 to SGs in response to various stressors. For example, thapsigargin, which perturbs intracellular calcium stores and is widely used to induce ER stress, was previously shown to induce TDP-43 recruitment to SGs in HeLa cells but not in Neuro2a cells [8], [15]. Whether or not modulation of TDP-43 recruitment to SGs has an effect on disease-relevant processes, such as inclusion formation, remains debated [14]. However, alterations in TDP-43 levels alter SG dynamics, suggesting that SG changes could occur in disease [8].

ER stress and induction of the unfolded protein response (UPR) are central to ALS pathophysiology [16]. When the UPR is induced three distinct signalling pathways are activated, mediated by inositol requiring kinase 1 (IRE1), activating transcription factor 6 (ATF6), and protein-kinase-like endoplasmic reticulum kinase (PERK) [17], [18], [19]. IRE1 activation leads to the splicing of X-box binding protein 1 (XBP-1) mRNA within the nucleus to produce a functional transcription factor. When ATF6 is activated, it is transported to the cis-Golgi compartment and is cleaved to produce an active transcription factor. In addition, activation of PERK causes general translational repression by stimulating SG formation via phosphorylation of eIF2α. Other consequences of UPR induction include up-regulation of ER chaperones, such as protein disulphide isomerise (PDI) [20]. Although initially protective, if unresolved, the UPR triggers apoptosis by ER stress-specific cell death signals, including induction of C/EBP-homologous protein (CHOP) via the PERK and ATF6 pathways [21], [22].

ER stress precedes the appearance of clinical features in ALS-linked mutant superoxide dismutase 1 (SOD1) transgenic rodents [23], and genetic manipulation of ER stress mediators modulates disease in these animals [24], [25]. ER stress is present in sporadic and familial forms of ALS, including those cases caused by mutations in fused in sarcoma (FUS), which bears structural and functional similarities to TDP-43 [23], [26], [27]. Increased genetic susceptibility to ER stress has also been linked with ALS [28]. Although TDP-43 is C-terminally fragmented and hyper-phosphorylated in disease [1], the factors which trigger these changes remain poorly defined. However, ER stress also causes TDP-43 fragmentation in cell culture [15], [29] and over-expression of TDP-43 causes changes in CHOP and XBP-1 signalling in cell culture and rat models of TDP-43-linked disease [30], [31].

The chaperone protein disulphide isomerase (PDI) is induced by ER stress and is up-regulated in human sporadic ALS and in animal models of mutant SOD1-linked ALS [23], [32], [33]. PDI may protect against ER stress, inclusion formation and cell death associated with mutant SOD1 expression by modulating abnormal disulphide bond formation [27], [34]. In addition, the cellular distribution of PDI in mutant SOD1 transgenic mice modifies disease processes [35] and PDI is a constituent of TDP-43-positive or FUS-positive inclusions found in motor neurons of ALS patients [26], [36]. Cross-linking of TDP-43 via disulphide bonds alters its conformation and function [37], suggesting that PDI is a potential candidate for proteins that interact with TDP-43 and prevent TDP-43 misfolding.

In this study we examined whether ER stress could act as a stressor that leads to cytoplasmic accumulation of TDP-43 and subsequent incorporation of TDP-43 into SGs. Six different ALS-linked TDP-43 mutants were examined: A315T and M337V, which have been reported in multiple familial ALS pedigrees [38], [39], [40], [41], [42], [43]; D169G, the only ALS-linked mutation identified that lies outside the C-terminal region [39]; and G294A, Q331K and N390D, which have been identified in sporadic ALS patients [39], [40]. Pharmacological induction of ER stress in cell culture led to cytoplasmic accumulation of wildtype TDP-43 and all six TDP-43 mutants. Furthermore, ER stress caused the rapid incorporation of TDP-43 into cytoplasmic SGs. This process was enhanced by pharmacological treatment with salubrinal to inhibit the deactivation of eIF2α, a key upstream modulator of ER stress. We also demonstrate that C-terminal TDP-43 fragments form inclusions in close association with PDI and the ER/Golgi apparatus, suggesting that TDP-43 inclusion formation causes dysfunction of ER/Golgi components. Moreover, over-expression of mutant TDP-43, and to a lesser extent wildtype TDP-43, induced ER stress via several UPR pathways, including activation of XBP-1 and ATF6, thus linking ER stress to neurodegeneration. Finally we show up-regulation of PDI in the spinal cords of transgenic mutant A315T TDP-43 mice, and interaction of mutant TDP-43 with PDI, providing further evidence of an ER-associated protective response in TDP-43 proteinopathies.

## Materials and Methods

### DNA constructs

Site-directed mutagenesis was performed to remove the stop codon and insert a unique *Bam*HI restriction enzyme site into a pcDNA3.1(+) Myc-tagged human TDP-43-encoding construct [4]. The Myc-TDP-43 sequence was inserted between *Hind*III and *Bam*HI restriction sites in pmCherry.N1 (Clontech), to allow expression of human TDP-43 with a C-terminal mCherry tag. The subsequent wildtype Myc-TDP-43-mCherry construct was used as a template to produce six ALS-linked mutant TDP-43 expressing vectors (D169G, G294A, A315T, Q331K, M337V, N390D) by QuikChange site-directed mutagenesis (Stratagene) according to the manufacturer's instructions. The 218–414 TDP-43-mCherry construct was produced by cloning of a PCR product from a primer incorporating a unique *Hind*III restriction enzyme site immediately 5′ to the M218 codon of TDP-43 into a pCR2.1-TOPO vector (Invitrogen) with subsequent sub-cloning into pmCherry.N1 using *Hind*III and *Bam*HI restriction sites. EGFP-TDP-43 constructs were as described previously [7], [44]. For the detection of ER stress, an ATF6-EGFP reporter construct (Addgene plasmid 32955) was used [45]. For co-immunoprecipitation experiments, a vector encoding PDI with a V5 epitope tag was used [46].

### Cell culture

Mouse neuroblastoma Neuro2a (ATCC cell line CCL-131), human epithelial HeLa (ATCC cell line CCL-2) and human embryonic kidney HEK293T cell lines were maintained in high glucose DMEM (Gibco) with 10% heat-inactivated fetal calf serum (Gibco), 100 µg/mL penicillin and 100 µg/mL streptomycin. Cells were treated as indicated with thapsigargin (Sigma) to induce ER stress, MG132 (Sigma) to inhibit proteasomal function, cycloheximide (Sigma) as a general translation inhibitor, and arsenite (Sigma) as a standard oxidative stress inducer of SGs. For immunocytochemistry, cells were plated on poly-L-lysine-coated 12 mm glass coverslips. Cells were transfected with constructs using Lipofectamine2000 with PLUS reagent (Invitrogen) according to the manufacturer's protocol. Cell lysates were collected in TN buffer (50 mM Tris-HCl pH 7.5 and 150 mM NaCl) with 0.1% SDS, 1% protease inhibitor cocktail (Sigma) and 1% phosphatase inhibitor (Sigma) by incubation on ice for 10 min. The supernatant was cleared by centrifugation at 16,100 *g* for 10 min (the SDS-soluble fraction). Protein concentrations of cell and tissue lysates were determined using the BCA protein assay (Thermo Scientific) by comparison with bovine serum albumin (BSA) standards.

### Immunocytochemistry and microscopy

Cells were fixed with 4% paraformaldehyde at room temperature for 15 min, permeabilised with 0.1% triton X-100 in PBS for 10 min and then blocked with blocking buffer (1% BSA in 0.03% triton X-100 in PBS) for 30 min at room temperature. Primary antibodies were diluted in blocking buffer and incubated overnight at 4°C. Primary antibodies were: mouse anti-PDI Ab2792 (Abcam), rabbit anti-TDP-43 10782 (Protein Tech Group), mouse anti-HuR 39-0600 (Invitrogen), rabbit anti-ERGIC53 E1031 (Sigma), rabbit anti-XBP-1 sc-7160 (Santa Cruz), mouse anti-GADD153/CHOP sc-7351 (Santa Cruz) and mouse anti-GM130 610823 (BD Transduction Labs). Cells were washed in PBS and then incubated with secondary antibody at 1∶5000 in PBS for 1 h at room temperature. Secondary antibodies used were: goat-anti-rabbit IgG AlexaFluor® 488/594, goat anti-mouse IgG AlexaFluor® 647 or rabbit-anti-mouse IgG AlexaFluor® 488 (all from Molecular Probes). Nuclei were stained using TO-PRO®-3 Iodide stain (Invitrogen), Hoechst 33342 or DAPI diluted 1∶2000-1∶10000 in PBS for 10 min. Images were acquired with constant gain and offset settings where appropriate using an Olympus Fluoview 1000 inverted confocal laser-scanning microscope or an Olympus IX81 inverted fluorescence microscope with Olympus digital camera. For SG quantification, 40X images of at least five random fields of view taken at ½ radius of the coverslip were used for analysis from at least three independent experiments. Cells bearing two or more HuR-positive and/or TDP-43-positive non-nuclear foci of greater than ∼1 µm in diameter were counted as cells containing SGs using ImageJ software. For detection of ER stress, 120 cells were counted in each group. An ATF6-GFP reporter construct was co-expressed with TDP-43 constructs and cells were immunostained for GM130 to visualise the Golgi apparatus. Cells in which ATF6 fluorescence co-localised with GM130 fluorescence, indicating translocation of ATF6 to the Golgi, were scored as having ER stress [45]. For detection of XBP-1 activation, cells were transfected with TDP-43 constructs and immunostained for XBP-1. Cells in which XBP-1 immunoreactivity was higher in the nucleus than the cytoplasm were scored as having ER stress, similar to previous studies [26], [47]. Likewise, cells with nuclear immunoreactivity for CHOP were scored as having ER stress, as performed previously [27].

### Transgenic mice

A315T TDP-43 transgenic mice were obtained from Jackson Laboratories and bred on a C57BL/6 background [48]. Experimental procedures and housing conditions for animals were approved by the University of Queensland and University of Melbourne Animal Ethics Committees. Spinal cords of three transgenic female mice at symptom onset (p92, p97 and p102) and age- and gender-matched non-transgenic littermate controls were analysed in triplicate by SDS-PAGE and immunoblotting. For immunohistochemistry, spinal cords of two male transgenic mice at p79 and p83 were analysed with matched non-transgenic littermates.

### SDS-PAGE and immunoblotting

Protein samples were electrophoresed through SDS-polyacrylamide gels and transferred to nitrocellulose membranes. Membranes were blocked with 5% skim-milk in TBS or 3% BSA in TBS-T for 30 min then incubated with primary antibodies at 4°C for 12–16 h. Primary antibodies used were: mouse anti-FLAG M2 (Sigma), mouse anti-β-actin AC-15 (Sigma), mouse anti-TDP-43 2E2-D3 (Abnova), rabbit anti-PDI SPA-890 (Stressgen), mouse anti-V5 R960-25 (Invitrogen) and rabbit anti-red fluorescent protein (RFP; Affinity Bioreagents). Membranes were incubated with secondary antibodies for 1 h at room temperature. Secondary antibodies used were: HRP-conjugated goat anti-rabbit AB132P or goat anti-mouse AB326P at 1∶10000 (both from Chemicon). Signals were detected using ECL reagent (Roche) with Biomax MR film (Kodak) or ChemiDoc imaging system (BioRad). Densitometry of immunoblots was performed using ImageJ software (NIH).

### Immunoprecipitation

Cell lysates (1 mg protein) at 24 hr post-transfection were incubated with anti-PDI or anti-RFP antibody or irrelevant, isotype-matched control antibody (anti-HA, Sigma) and 100 µl 50% (wt/vol) protein A-Sepharose CL-4B (Amersham Biosciences) in TN buffer with 0.5% NP40 and incubated on a rotating wheel overnight at 4°C. Samples were centrifuged at 2000 *g* for 2 min, and Sepharose pellets were washed twice in TN buffer + NP40. For immunoblotting, immunoprecipitates were liberated by boiling in 4× SDS sample buffer with 20% β-mercaptoethanol.

### Statistical analyses

Data are presented as mean ± standard error of the mean (SEM) from at least three independent experiments and were analysed by unpaired t-test or ANOVA followed by Bonferonni's post-test unless otherwise stated. p<0.05 was considered statistically significant.

## Results

### Sub-cellular location of wildtype and mutant TDP-43-mCherry proteins

**Figure 1:**
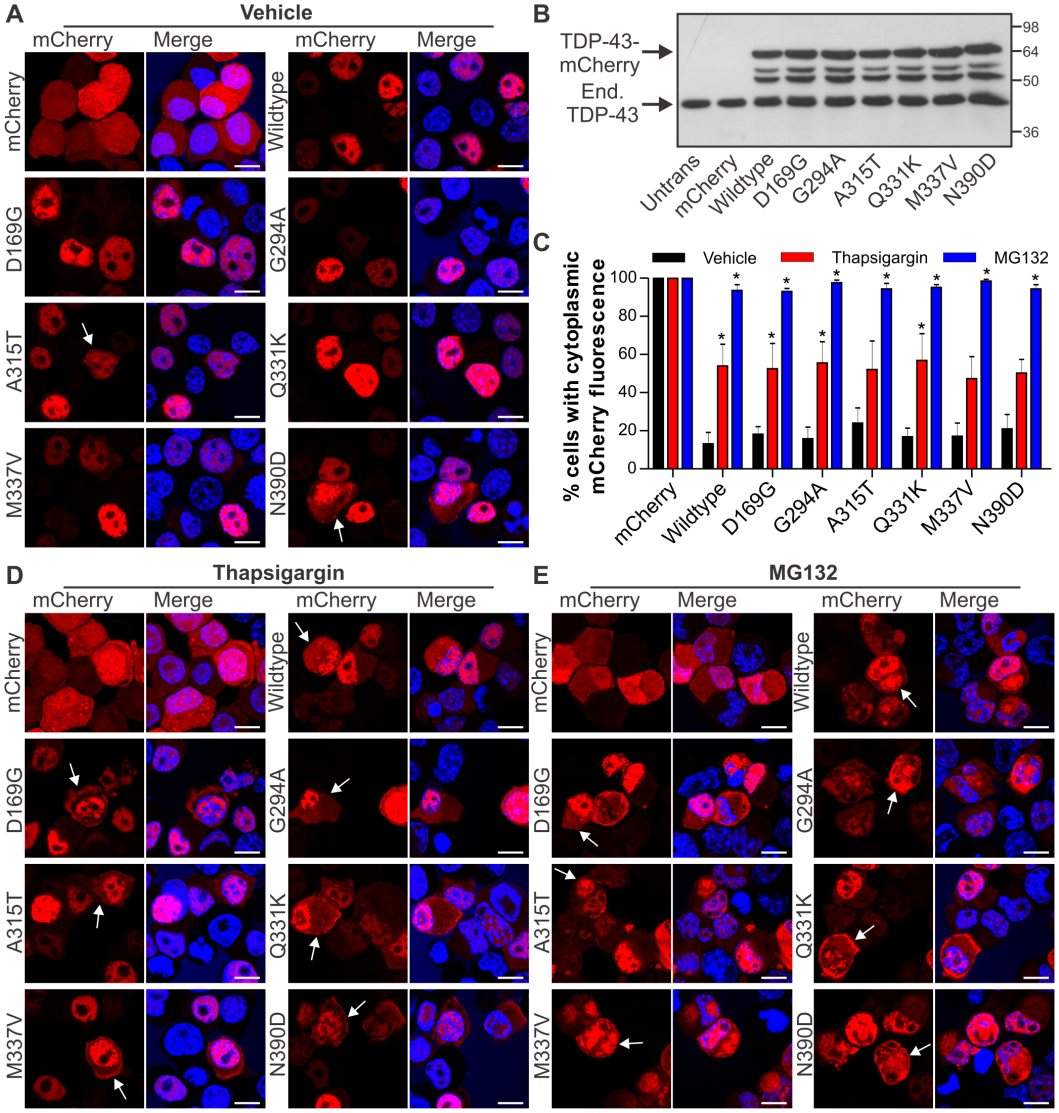
ER stress and proteasome inhibition cause redistribution of wildtype and ALS-linked mutant TDP-43. (**A**) Neuro2a cells were transfected with the construct indicated to the left of each series of panels (mCherry alone, wildtype TDP-43-mCherry, or mutant D169G, G294A, A315T, Q331K, M337V or N390D TDP-43-mCherry), and fixed at 48 h post-transfection. mCherry fluorescence is shown in the left panels, and Hoechst nuclei stain is shown in the merged right panels. Note the largely nuclear distribution of wildtype and mutant TDP-43-mCherry, but with low levels of non-nuclear mCherry fluorescence in some cells, indicated by arrows. (**B**) Immunoblot of cell lysates expressing mCherry alone or wildtype or mutant TDP-43-mCherry showing equal expression levels of all proteins, and levels of endogenous (End.) TDP-43. Approximate molecular weight markers are shown on the right. (**C**) Quantification of the effect of thapsigargin or MG132 treatment on the percentage of Neuro2a cells with cytoplasmic mCherry fluorescence. Results are expressed as mean ± SEM, *n* = 3, *p<0.05 versus respective vehicle treated controls by two-way ANOVA with Bonferroni's post-test. (**D**) Neuro2a cells were transfected as in **A** and treated with 100 nM thapsigargin for 24 h prior to fixation. (**E**) Neuro2a cells were transfected as in **A** and treated with 10 µM MG132 for 24 h prior to fixation. Note the increased non-nuclear distribution of wildtype and mutant TDP-43-mCherry with both thapsigargin and MG132 treatment, indicated by arrows. All scale bars represent 10 µm.

Six TDP-43 mutations previously linked to ALS were chosen for investigation in this study: A315T, M337V, D169G, G294A, Q331K and N390D [38], [39], [40], [41], [42], [43]. The cellular distribution of mCherry tagged proteins was analysed in Neuro2a cells using confocal microscopy. In contrast to the diffuse cytoplasmic staining of mCherry, transiently transfected TDP-43-mCherry constructs resulted in punctate fluorescence confined to, but dispersed throughout, the nucleus with exclusion from nucleoli (Fig. 1A) This distribution is similar to previous observations for endogenous TDP-43 [5], [49], [50] and confirms correct targeting of the fusion proteins (Fig. 1A).

There was moderate cytoplasmic fluorescence in approximately 10–20% of cells expressing either wildtype or mutant TDP-43-mCherry (Fig. 1C). The sub-cellular distribution of the wildtype and mutant proteins were similar and inclusions were not observed. Wildtype and mutant TDP-43-mCherry proteins (∼70 kDa) were detected at levels similar to the endogenous protein with a transfection efficiency of approximate 50%, suggesting an approximate two-fold expression level of the fusion protein compared to endogenous in transfected cells (Fig. 1B).

### ER stress causes redistribution of TDP-43 from the nucleus to the cytoplasm

ER stress was chronically induced in Neuro2a cells expressing TDP-43–mCherry by treating with 100 nM thapsigargin for 24 h. The cells were fixed 48 h after transfection, and examined by confocal fluorescence microscopy. Cytoplasmic TDP-43-mCherry was detected in approximately 50% of wildtype and mutant TDP-43 expressing cells treated with thapsigargin, compared with 10–20% of untreated cells (Fig. 1D). In comparison, cytoplasmic TDP-43-mCherry fluorescence was observed in almost all cells (95%) treated for 24 h with 10 µM MG132, a proteasome inhibitor [51] (Fig. 1E). Neither treatment caused cell death, and TDP-43-mCherry-positive inclusions were not observed in any of the conditions examined. Hence both ER stress and proteasome inhibition result in accumulation of both wildtype and mutant TDP-43 in the cytoplasm of cells without inclusion formation.

### ER stress causes TDP-43-positive stress granule formation which is enhanced by salubrinal pre-treatment

SG formation was identified by immunoreactivity to antibodies against the SG component Hu-antigen R (HuR) [11] in HeLa cells, in which SGs can be readily visualised due to cell morphology. Cells were pre-treated with either salubrinal (50 µM) or cycloheximide (50 µg/mL) and then either ER stress (using thapsigargin) or oxidative stress (using arsenite) was acutely induced, with appropriate vehicle-treated controls in both pre-treatment and stress conditions. The purpose of salubrinal pre-treatment was to potentiate induction of the PERK-mediated ER stress pathway by inhibiting eIF2α dephosphorylation [52], whereas cycloheximide, which prevents SG formation by inhibiting translation, was used as a negative control.

**Figure 2:**
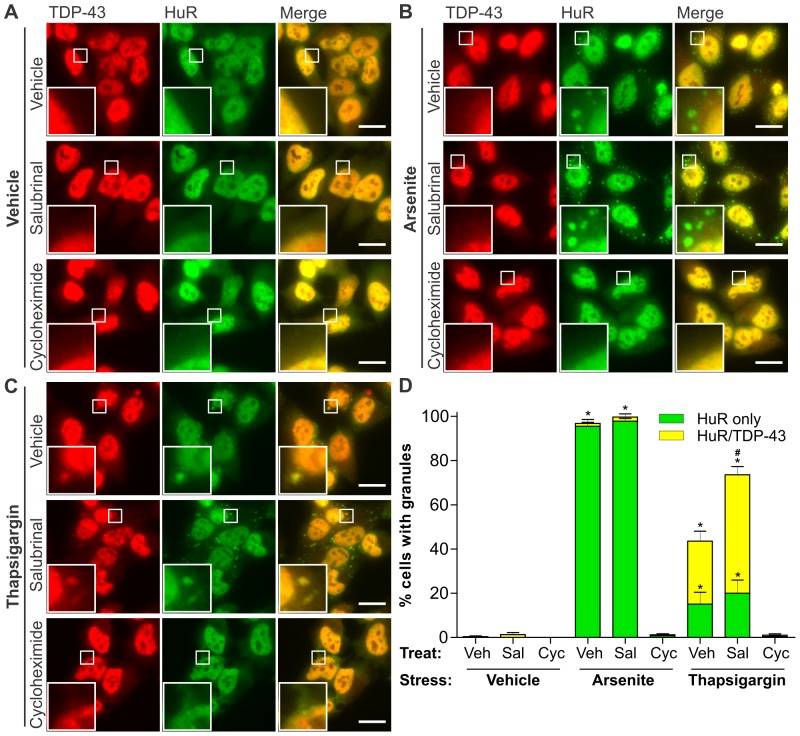
Formation of ER stress-induced TDP-43-positive stress granules is enhanced by salubrinal pre-treatment. HeLa cells were pre-treated for 2 h with either vehicle control, 50 µM salubrinal or 50 µg/mL cycloheximide and then stressed for an additional 1 h by treatment with vehicle control (**A**), 0.5 mM arsenite (**B**), or 10 µM thapsigargin (**C**) in the presence of the respective pre-treatment conditions. Cells were processed for immunocytochemistry using antibodies against TDP-43 (left panels) and HuR (middle panels). Merged images are shown on the right. Boxed regions in the main panels are shown magnified in the bottom left of each panel. (**D**) Quantification of the effect of aresenite or thapsigargin in the presence of salubrinal or cycloheximide on the formation of HuR-positive or HuR/TDP-43-positive cytoplasmic SGs. Results are expressed as mean ± SEM, *n* = 3, *p<0.05 versus respective vehicle-treated controls, #p<0.05 versus vehicle pre-treated/thapsigargin treated control, by two-way ANOVA followed by Bonferroni's post-test. All scale bars represent 20 µm.

Both TDP-43 and HuR were located primarily in the nucleus with no evident cytoplasmic puncta in the vast majority of vehicle treated cells regardless of pre-treatment with either vehicle control, 50 µM salubrinal or 50 µg/mL cycloheximide (Fig. 2A). Treatment with arsenite, which is a typical SG inducer, caused HuR-positive SG formation in 95–100% of vehicle or salubrinal pre-treated cells, which was completely inhibited by cycloheximide pre-treatment (Fig. 2B). However, TDP-43-positive SGs were rarely observed in arsenite-treated cells under these conditions (Fig. 2B, D), similar to previous findings in HEK293 cells [9]. Cytoplasmic puncta positive for TDP-43 but negative for HuR were rarely observed under any of the treatment conditions used. Salubrinal pre-treatment did not alter the percentage of cells bearing SGs or the percentage of TDP-43-positive SGs following arsenite-induced oxidative stress.

Next, the role of ER stress in SG assembly was investigated by acutely treating vehicle-, salubrinal- or cycloheximide-pre-treated cells with 10 µM thapsigargin for 1 h, which is consistent with conditions reported by others previously to induce SGs [53], [54]. Approximately 20% of cells showed HuR-positive/TDP-43-negative SGs in both vehicle- or salubrinal-pre-treated conditions upon thapsigargin treatment, whereas SGs were not formed in thapsigargin treated cells pre-treated with cycloheximide (Fig. 2C, D). Additionally, thapsigargin treatment caused 20–30% of vehicle-pre-treated cells to form SGs positive for both HuR and TDP-43. Furthermore, salubrinal pre-treatment significantly increased the percentage of cells with HuR-positive/TDP-43-positive SGs to approximately 50%, but did not change the percentage of cells bearing HuR-positive/TDP-43-negative SGs, upon thapsigargin treatment (Fig. 2D). Overall, salubrinal pre-treatment increased the percentage of thapsigargin treated cells bearing HuR-positive SGs (with or without TDP-43) from ∼40% in controls to ∼75% (Fig. 2D).

These results demonstrate that ER stress is a potent inducer of TDP-43 recruitment to SGs, and that inhibiting eIF2α dephosphorylation with salubrinal specifically enhances the formation of TDP-43-positive SGs. Recruitment of TDP-43 to cytoplasmic SGs appeared to be specifically related to ER stress since treatment with arsenite to induce oxidative stress did not cause TDP-43 redistribution to SGs under the conditions used in these experiments.

### C-terminal TDP-43 forms peri-nuclear inclusions that associate with markers of the ER-Golgi compartments

**Figure 3:**
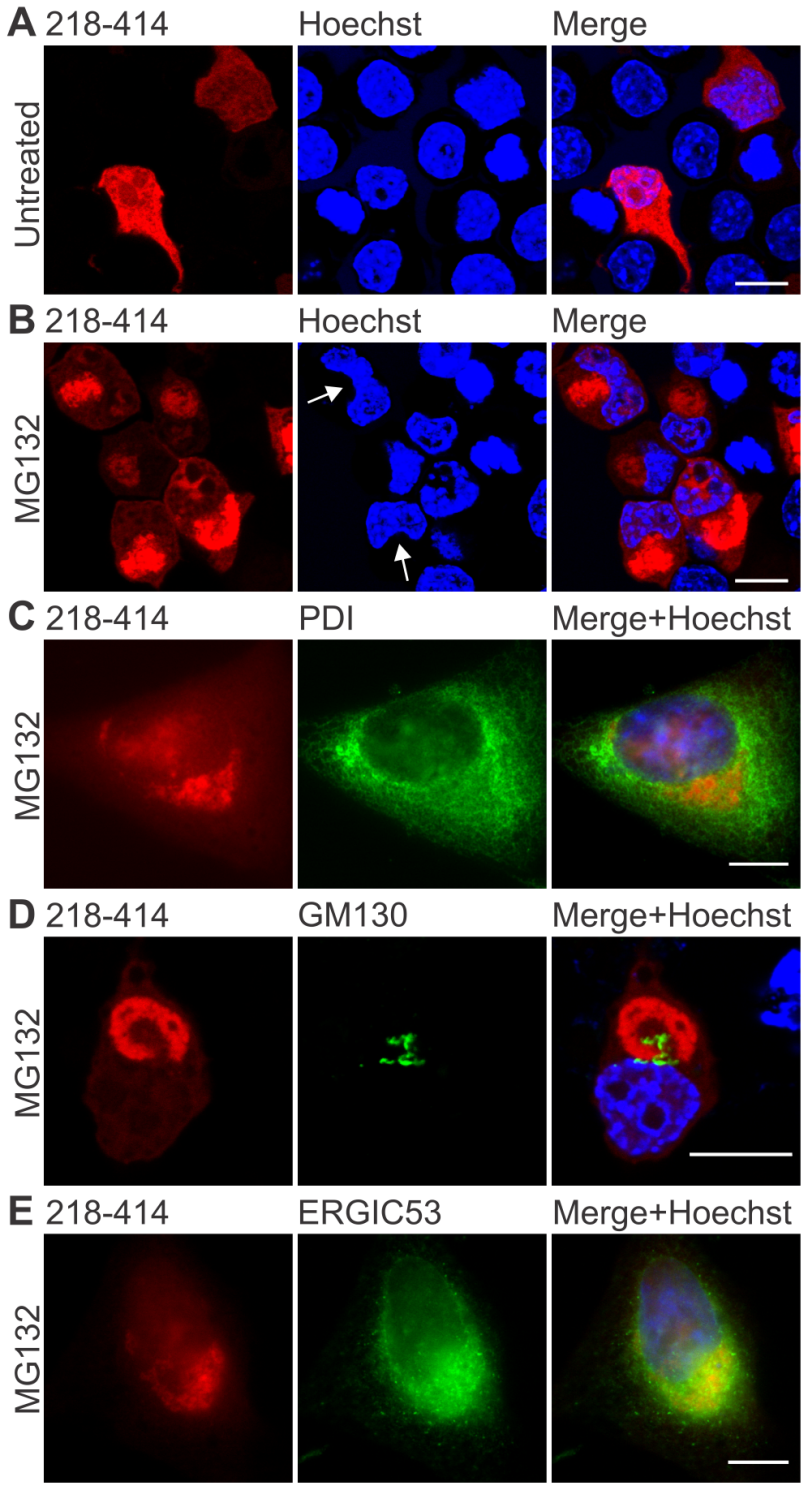
C-terminal 218–414 TDP-43 inclusions form in association with the Golgi and ER. (**A, B**) Expression of wildtype 218–414 TDP-43-mCherry in Neuro2a cells, with mCherry fluorescence (left images), Hoechst nuclei stain (middle images), and merged images (right). Panel **A** shows untreated cells, and panel **B** shows cells treated with 10 µM MG132 for 24 h. Arrows indicate nuclei showing distorted morphology, due to the presence of inclusions. Note that the mCherry fluorescence image shown in **A** was captured using increased gain and offset settings compared to that shown in **B** in order to allow detection. (**C**) MG132-treated HeLa cells expressing 218–414 TDP-43-mCherry (red, left image), with immunocytochemistry for PDI (green, middle image). (**D**) Neuro2a cells were transfected and treated as in **B** and processed for immunocytochemistry against GM130 (green, middle image). In MG132-treated HeLa cells expressing 218-414 TDP-43-mCherry (red, left image), immunocytochemistry is shown for (**E**) ERGIC53 (green, middle image). Merged images shown with Hoechst nuclei stain are on the right. All scale bars represent 10 µm.

**Figure 4:**
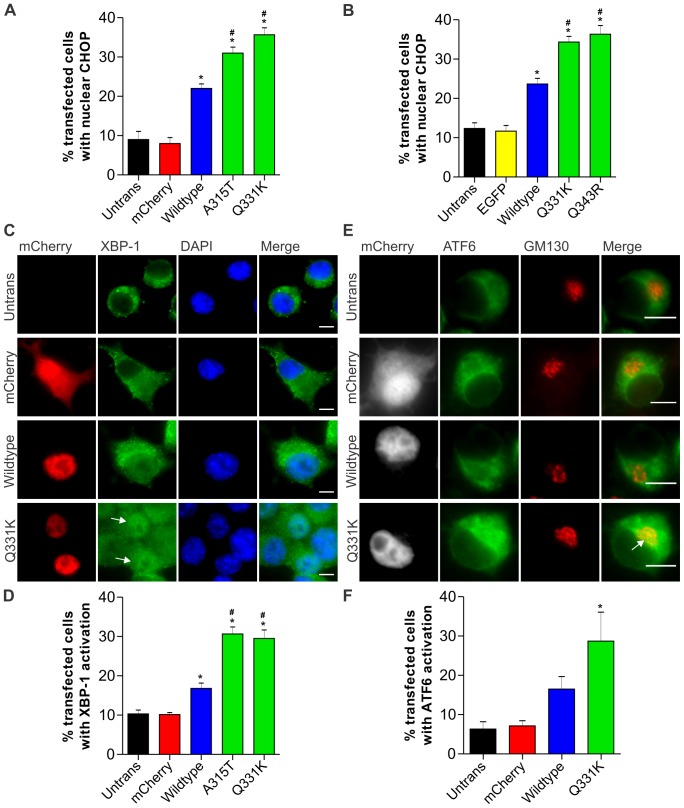
Wildtype and ALS-linked mutant TDP-43 proteins induce ER stress. (**A**) Increase in nuclear CHOP immunoreactivity in Neuro2a cells expressing wildtype and ALS mutant forms of TDP-43 linked with mCherry. (**B**) Increase in nuclear CHOP immunoreactivity in Neuro2a cells expressing wildtype and ALS mutant forms of TDP-43 linked with EGFP. (**C**) XBP-1 is activated in mutant TDP-43 Q331K cells, indicating induction of the IRE1 pathway of ER stress. Cells expressing TDP-43 mCherry (first column, red) are shown with XBP-1 (second column, green) and DAPI staining (third column, blue). Merge (fourth column) indicates overlays of the fluorescent images of XBP-1 and DAPI. Arrow indicates cells with increased nuclear XBP-1. Scale bars represent 5 µm. (**D**) Quantification of transfected cells with activation of XBP-1, as determined by an increase in nuclear XBP-1 immunoreactivity. (**E**) ATF6 translocates to the Golgi in mutant Q331K cells, indicating activation of the ATF6 pathway of ER stress. Neuro2a cells were co-transfected with mCherry constructs (first column, white) and ATF6-GFP (second column, green). Cells were fixed and immunostained for Golgi marker GM130 (third column, red). Merge (fourth column) indicates overlays of the fluorescent images of ATF6 and GM130. Arrow indicates co-localisation of ATF6 in the Golgi apparatus. Scale bars represent 10 µm. (**F**) Quantification of transfected cells with activation of ATF6, as determined by co-localisation of ATF6 with GM130. Data are represented as mean ± SEM; *p<0.05 versus mCherry or EGFP controls and #p<0.05 versus respective wildtype TDP-43 proteins, by one-way ANOVA with Tukey's post hoc test.

The sub-cellular positioning, relative to ER-Golgi proteins, of SGs formed by endogenous TDP-43 in thapsigargin-stressed was examined in HeLa cells, because their flat extended morphology lends itself to the examination of protein co-location using confocal microscopy. The TDP-43-positive SGs did not co-locate with PDI or markers of the ER or the Golgi apparatus (KDEL and ERGIC-53), implying that they form in the cytoplasm and outside of the ER-Golgi system (data not shown). We next produced a vector to allow expression of mCherry-tagged TDP-43 residues 218-414 (Fig. 3), since full-length wildtype or mutant TDP-43-mCherry did not form inclusions when expressed in several mouse or human cell lines (Fig. 1, Fig. 4 and data not shown). TDP-43 residues 218-414 correspond to the potentially pathogenic ∼20 kDa caspase cleavage product previously shown to be a highly aggregate-prone species of TDP-43 [6], [44], [55].

Under basal conditions, the expression of 218–414 TDP-43-mCherry could not be detected by immunoblotting up to 72 h after transient transfection in Neuro2a cells, however diffuse cytoplasmic mCherry fluorescence was evident under confocal microscopy at very low levels in a small proportion of cells (Fig. 3A). In contrast, approximately 70% of cells treated with the proteasome inhibitor MG132 formed large juxta-nuclear inclusions that partially displaced the nucleus and were reminiscent of inclusions seen in human pathology (Fig. 3B). These data indicate that in cell culture, 218–414 TDP-43-mCherry is degraded rapidly by the proteasome, but that large inclusions form when the proteasome is inhibited.

Neuro2a or HeLa cells were transiently transfected with 218–414 TDP-43-mCherry, treated with MG132 and the sub-cellular location of 218–414 TDP-43-mCherry inclusions, relative to PDI and other proteins of the ER and Golgi apparatus was examined by immunocytochemistry. Small peri-nuclear inclusions accompanied by one or two larger juxta-nuclear inclusions were observed in most cells (Fig. 3C–E). These inclusions were surrounded by PDI, which is predominately found in the ER, consistent with a recent report describing the co-location of PDI with TDP-43 in sporadic ALS tissues (Fig. 3C) [36]. The relative locations of 218–414 TDP-43-mCherry inclusion and markers of the Golgi apparatus and ER-Golgi intermediate compartment (ERGIC, a distinct organelle which mediates trafficking between the ER and Golgi apparatus) was then examined. The large juxta-nuclear inclusions formed in close association with the cis-Golgi marker GM130 (Fig. 3D), often surrounding regions of GM130 immunoreactivity (Video S1). The 218–414 TDP-43-mCherry inclusions also showed partial co-location with the ERGIC marker, ERGIC-53 (Fig. 3E). Overall, these results indicate that 218–414 TDP-43 inclusions form adjacent to the nucleus and in close association with the Golgi and ERGIC when the proteasome is inhibited. The association between inclusions and TDP-43 pathology thus indicates possible disturbance of ER-Golgi transport function in ALS.

### Over-expression of mutant TDP-43 induces ER stress to a greater extent than wildtype TDP-43

In the well-studied model of ALS induced by mutant forms of SOD1, ER stress and UPR induction precede inclusion formation [27], [32], [56]. Hence we asked whether wildtype and mutant TDP-43 can induce ER stress in cell culture. CHOP is a pro-apoptotic protein induced predominately via the PERK and ATF6 UPR pathways and its levels are a useful marker of UPR induction. We observed that at 18–24 h post-transfection, a greater proportion of Neuro2a cells demonstrated cytoplasmic rather than nuclear TDP-43, whereas after 24 h TDP-43 localised predominately in the nucleus. Hence we examined cells for induction of ER stress at 18 h post-transfection, CHOP levels in Neuro2a cells, measured by immunocytochemistry, were slightly but significantly greater in cells expressing wildtype TDP-43-mCherry compared to mCherry alone. Furthermore, CHOP levels in cells expressing two ALS-linked TDP-43 mutants, A315T or Q331K, were dramatically increased compared to both mCherry alone or wildtype TDP-43-mCherry (Fig. 4A). This was subsequently confirmed using EGFP-tagged TDP-43 Q331K and another ALS mutant, Q343R, showing increased CHOP activation in mutant expressing cells compared to control cells expressing EGFP alone or wildtype EGFP-TDP-43 (Fig. 4A–B).

Others have made the observation that CHOP, but no other UPR marker, is up-regulated following viral overexpression of wildtype TDP-43 in cell culture [31]. However, we examined additional markers of ER stress in Neuro2a cells using single-cell immunocytochemical analysis, which allows analysis of only transfected cells rather than analysis of the entire population as occurs using immunoblotting techniques [26], [27], [57], [58]. IRE1 activation was measured by detection of nuclear XBP-1. Over-expression of wildtype TDP-43-mCherry slightly but significantly increased XBP-1 activation compared to mCherry expressing cells (Fig. 4C–D). Furthermore, XBP-1 activation was dramatically increased by expression of A315T or Q331K mutant TDP-43-mCherry compared to both mCherry alone or wildtype TDP-43-mCherry. Finally, over-expression of wildtype TDP-43-mCherry slightly increased activation of ATF6 compared to mCherry expressing cells, although this did not reach statistical significance. However, over-expression of Q331K mutant TDP-43-mCherry significantly increased activation of ATF6 (Fig. 4E–F). These data indicate that ALS-linked mutant TDP-43 proteins activate the major signalling pathways of ER stress. Furthermore, over-expression of wildtype TDP-43 also induces ER stress, but to a lesser extent than mutant TDP-43.

### PDI co-immunoprecipitates with mutant TDP-43 and is up-regulated in transgenic A315T mutant TDP-43 mouse spinal cord

**Figure 5:**
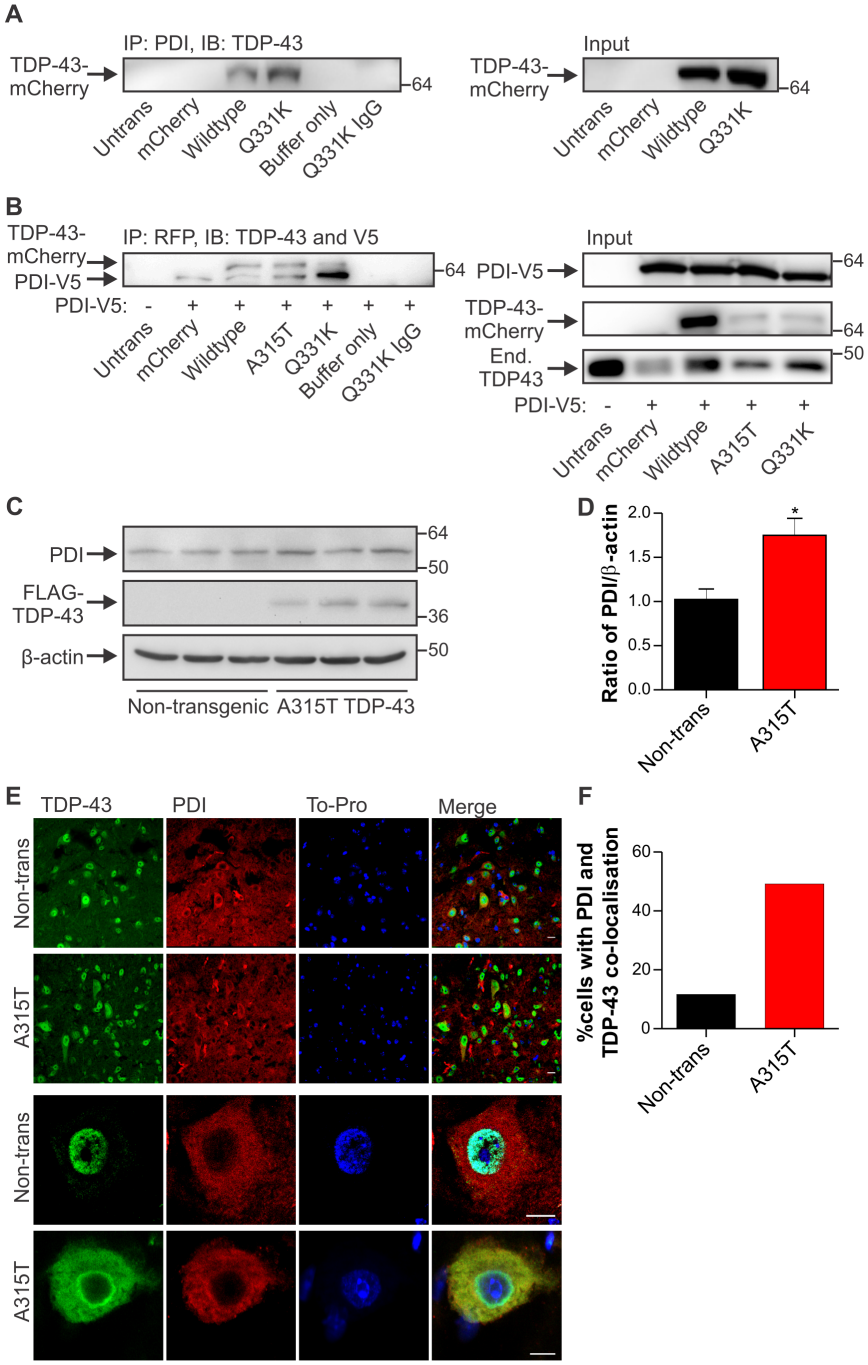
PDI co-precipitates with TDP-43 and is increased in mutant TDP-43 transgenic mouse spinal cords. (**A**) Co-immunoprecipitation of wildtype and mutant Q331K TDP-43-mCherry from Neuro2a cell lysates using an anti-PDI antibody followed by TDP-43 immunoblot. Control immunoprecipitations were performed using untransfected (Untrans) or mCherry expressing cell lysates, buffer only with coprecipitating antibodies or TDP-43 Q331K cell lysates using an irrelevant, isotype-matched control antibody (IgG). Input control (2%) shows expression of mCherry-TDP43 in wildtype and Q331K cell lysates. (**B**) Co-immunoprecipitation of PDI-V5 from wildype and mutant A315T and Q331K TDP-43-mCherry from HEK293T cell lysates using an anti-RFP antibody to precipitate mCherry followed by TDP-43 and V5 immunoblot. Control immunoprecipitations were performed using Untrans cell lysates or mCherry and PDI-V5 expressing cell lysates, buffer only with coprecipitating antibody or Q331K TDP-43 and PDI-V5 co-transfected cell lysates using an irrelevant, isotype-matched control antibody (IgG). Input control shows expression of PDI-V5, TDP-43-mCherry and endogenous (End.) TDP-43. Approximate molecular weight markers are shown on the right, and representative images are shown. (**C**) Proteins were extracted from spinal cords of three mutant A315T TDP-43 transgenic mice and three litter-matched non-transgenic controls, and immunoblotting was performed for PDI, FLAG-TDP-43 (detected using antibody against FLAG), and β-actin. (**D**) Quantification of PDI levels from immunoblots normalised to β-actin by densitometry. Data represent mean normalised values from three independent analyses and are shown as mean ± SEM. *p<0.05 versus non-transgenic controls by unpaired two-tailed t-test. (**E**) Immunohistochemistry for TDP-43 and PDI in non-transgenic and A315T mutant TDP-43 mouse spinal cords. Cells were immunostained for TDP-43 (first column, green), PDI (second column, red), and were stained using To-Pro to identify nuclei (third column, blue). Merged images (fourth column) are also shown. Increased co-localisation of PDI with TDP-43 is seen in A315T animals compared to non-transgenic controls (Non-trans). All scale bars represent 10 µm. (**F**) Quantification of the percentage of motor neurons in which PDI and TDP-43 were co-localised. A total of 60 cells per group were counted, and data represent mean values from two mice per group.

We showed that mutant TDP-43 induces ER stress and since abnormal TDP-43 disulphide bonding occurs in FTLD [37], we next examined the possibility that the ER disulphide-modulating chaperone PDI, which was previously implicated in mutant SOD1-linked and sporadic ALS [23], [32], was also involved in TDP-43-linked disease. Immunoprecipitation was used to examine interactions between PDI and TDP-43. Lysates from Neuro2a cells transfected with TDP-43-mCherry constructs were immunoprecipitated using an anti-PDI antibody, and immunoblot analysis revealed that mutant Q331K TDP-43, and to a lesser extent wildtype TDP-43, co-precipitated with PDI (Fig. 5A). Control reactions containing buffer only, untransfected cells or precipitation of Q331K TDP-43 cell lysates using an irrelevant, isotype-matched control antibody were negative, demonstrating a specific interaction between PDI and TDP-43. To confirm the interaction, we also performed co-immunoprecipitaion experiments using HEK293T cells co-transfected with TDP-43-mCherry and PDI-V5 encoding constructs using an anti-RFP antibody to bind the mCherry tag, followed by immunoblotting for V5, to detect over-expressed PDI-V5, and TDP-43, to confirm immunoprecipitation with the RFP antibody. PDI-V5 was markedly co-immunoprecipitated with mutant A315T and Q331K TDP-43-mCherry, and with wildtype TDP-43-mCherry and mCherry alone to a lesser extent (Fig. 5B). Control reactions containing buffer only, untransfected cells or precipitation of Q331K TDP-43 and PDI-V5 over-expressing cell lysates using an irrelevant, isotype-matched control antibody were negative (Fig. 5B).

The involvement of PDI in an *in vivo* model of TDP-43 proteinopathy was then examined using spinal cord tissues from transgenic A315T mutant TDP-43 mice [48]. By immunoblotting, PDI levels were modestly but significantly higher in A315T TDP-43 mouse spinal cords at disease onset (approximately p90–100) than in age, gender and litter-matched non-transgenic controls (Fig. 5C–D). When assessed by immunohistochemistry, PDI was more widely distributed in the spinal cord of transgenic A315T TDP-43 mice just prior to disease onset, compared to non-transgenic controls (Fig. 5E). PDI and TDP-43 were co-localised in a greater proportion of motor neurons in A315T TDP-43 mice than in non-transgenic controls (Fig 5F).

## Discussion

**Figure 6:**
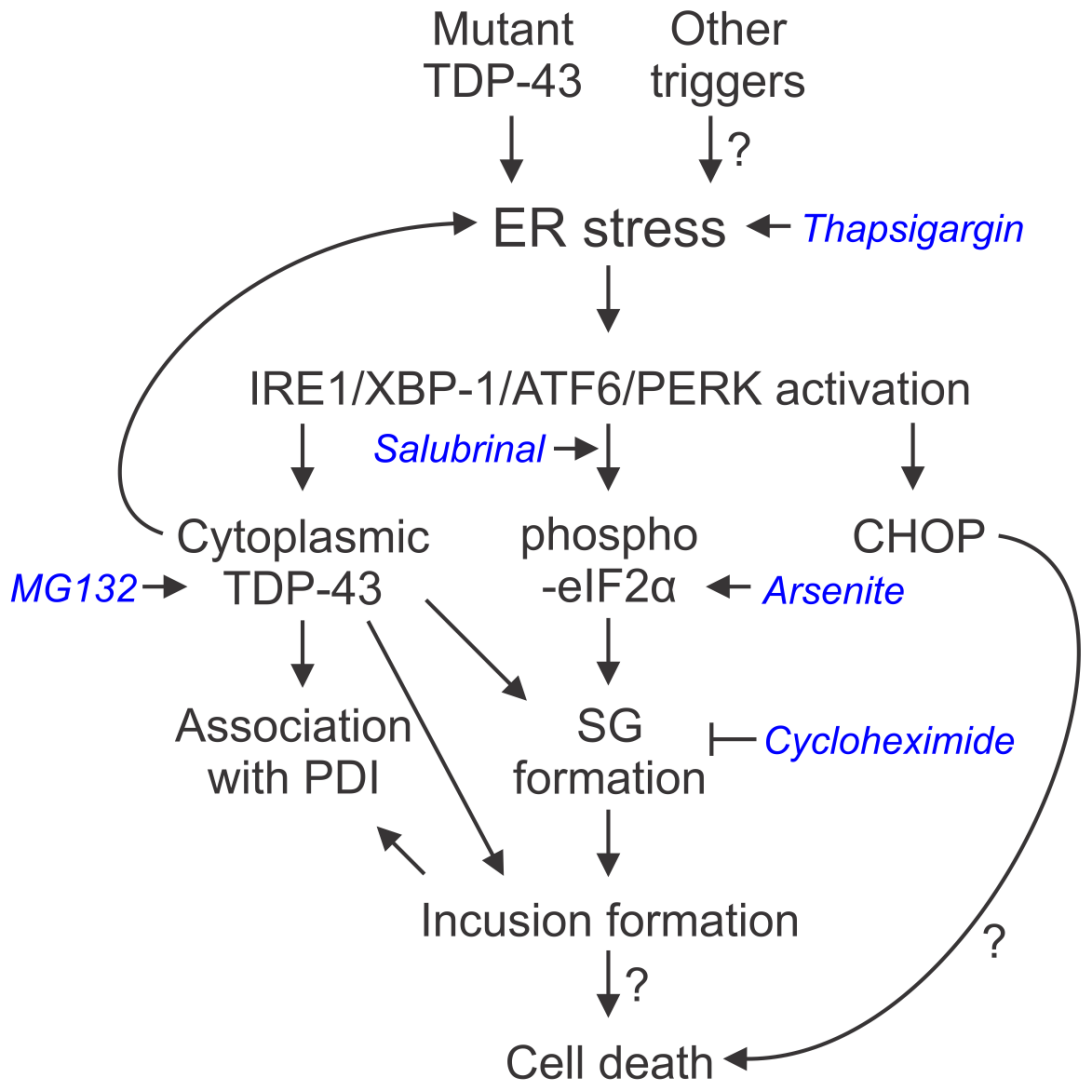
A model for the involvement of ER stress in TDP-43 proteinopathies. ER stress activation causes accumulation of cytoplasmic TDP-43, and induction of TDP-43-positive SGs, which could lead to inclusion formation and neuron death in disease. ER stress may also lead to cell death via apoptotic signalling involving the transcription factor CHOP, independent of SGs and inclusion formation. Additionally, TDP-43 shows increased association with the chaperone PDI in disease. Pharmacological agents used in this study are shown italicised in blue.

In this study, we demonstrate that ER stress leads to accumulation of both wildtype and ALS-linked TDP-43 mutants in the cytoplasm and to incorporation of TDP-43 into cytoplasmic SGs, We also demonstrate that C-terminal TDP-43 inclusions, induced by proteasome inhibition, are closely associated with PDI and the ER-Golgi compartments, suggesting potential disturbances to these organelles in TDP-43-linked disease. Indeed, over-expression of wildtype TDP-43, and to an even greater extent ALS-linked mutant TDP-43, induced ER stress via multiple ER stress signalling pathways. Also, PDI, a chaperone induced during ER stress, interacted with TDP-43 and co-located with TDP-43 in the spinal cords of transgenic mutant A315T TDP-43 mice, consistent with the increasing evidence that alterations of PDI location and function are common features in ALS. These results suggest that ER stress is involved in modulating TDP-43 sub-cellular distribution, with potential implications for triggering pathology and neuronal death in TDP-43 proteinopathies (Fig. 6).

It is well established that ER stress is an upstream event in ALS and this study confirms that ALS-associated mutant TDP-43 joins the list of disease proteins that induce ER stress. ER stress is a feature of sporadic human ALS [23], [27], in familial forms of ALS and FTLD, including those linked to FUS [26], vesicle-associated membrane protein-associated protein B (VAPB) [59] and valosin containing protein [60], and in mutant SOD1 animal and cell models [23], [24], [25], [61]. As TDP-43 is the major pathological protein in most cases of ALS and FTLD, ER stress may be a trigger for TDP-43 dysfunction. This is supported by studies demonstrating fragmentation of TDP-43, another pathological feature of disease, following induction of ER stress in cell culture [15], [29].

We found that transfection with ALS-linked mutant TDP-43, and to a lesser extent wildtype TDP-43, increased ATF6 and XBP-1 activation and induced CHOP as markers of ER stress, at 18–24 h post-transfection. However at later time points the induction of ER stress was variable or not detected (data not shown). The reason for this is unclear but may be linked to accumulation of TDP-43 in the cytoplasm, which we found to vary considerably depending on time after transfection. This may explain the findings of a recent study which reported only up-regulation of CHOP, but not other markers of ER stress, in NSC-34 cells with viral over-expression of wildtype TDP-43 at 24–48 hr post-infection [31]. We recently found that cytoplasmic localisation of mutant FUS was required for ER stress induction, whereas cells with only nuclear FUS had undetectable levels of ER stress [26]. Hence it is possible that similar to FUS, ER stress is closely linked to, but precedes, accumulation of TDP-43 in the cytoplasm. Abnormal activation of XBP-1 has also been described in a rat model with over-expression of mutant TDP-43, in which neurons display an unexpected decrease in XBP-1 levels while microglia display increased XBP-1 [30]. Combined, these studies suggest a non-classical ER stress response in TDP-43-linked disease.

We also interpret our findings as showing that ER stress is a key pathway in the formation of eIF2α-mediated SGs and the incorporation of TDP-43 into these SGs. Salubrinal, which inhibits dephosphorylation of eIF2α and potentiates the PERK pathway of ER stress, markedly increased TDP-43-positive SG formation following thapsigargin treatment. This argues that ER stress induces SGs via the eIF2α-mediated pathway. Indeed, ER stress activates PERK to inhibit protein translation, which is also the primary consequence of SG formation. The PERK arm of the UPR may be a defence against ALS because salubrinal is protective in a mutant SOD1^G93A^ mouse model of ALS [61], and disease was accelerated in SOD1^G85R^ mice with hemizygous deletion of PERK [25]. Interestingly, under basal conditions, prevention of eIF2α dephosphorylation alone is not sufficient to induce TDP-43 SG formation because the cellular stress of adding thapsagargin was required for SG formation and TDP-43 incorporation into SGs (Fig. 2). These results also show that ER stress is an important precursor to both SG formation and TDP-43 dysfunction in ALS. Previous findings showing ER stress-mediated cell death in mutant SOD1 models of ALS [24], [57] suggest that ER stress could be similarly involved not only in SG formation and TDP-43 redistribution but also in neuron death in TDP-43-linked disease. Indeed, recent studies have shown a protective effect of salubrinal treatment in a *C. elegans* model of TDP-43 proteinopathy [62].

It remains to be determined how different cellular stressors are able to differentially modulate TDP-43 redistribution into SGs. Previous studies have been conflicting in the effect of arsenite, a typical SG trigger, to form TDP-43 SGs. Some studies have indicated that arsenite does cause TDP-43 SG formation [8], [63], while others have shown a lesser effect [9], [37]. Our findings suggest that arsenite is not a strong inducer of TDP-43-positive SGs in HeLa cells under the conditions used here. Additionally, we show that salubrinal had no additive effect on the incorporation of TDP-43 into SGs following arsenite treatment, in contrast to the potentiation of TDP-43-positive SG formation with salubrinal treatment of cells under thapsigargin-induced ER stress. These results suggest differences in the mechanism of SG formation under different stress conditions, indicating potential differences in phosphorylation of eIF2α under different stress conditions, and highlighting the important role for ER stress in TDP-43 SG formation. Further investigation is warranted of the role of ER stress in TDP-43 accumulation and SG formation in neurons and *in vivo*.

We previously provided evidence that PDI can attenuate the effects of mutant SOD1 in cell models of ALS [27] and that PDI is up-regulated in sporadic human ALS spinal cord tissue [23]. Our findings here support other studies showing that PDI co-localisation with TDP-43-positive inclusions in sporadic ALS motor neurons [36], although the lack of full-length TDP-43 inclusions in our models makes direct comparison with human disease difficult. Thus TDP-43 can be added to the growing list of proteins linked to ALS that associate with PDI, including mutant SOD1 [32], FUS [26], and VAPB [64]. The co-immunoprecipitation of PDI with mutant TDP-43, and to a lesser extent wildtype TDP-43, in cell lysates implies that TDP-43 and PDI physically interact, suggesting that PDI is therefore in a position to protect against TDP-43 misfolding and aggregation. PDI is responsible for the isomerisation of protein disulphide bonds, forming native structures, and it is also a general protein chaperone. Cysteine oxidation and cross-linking of TDP-43 via disulphide bonds can alter conformations and function [37] and might induce aggregation of TDP-43 by a mechanism similar to that proposed for mutant SOD1 involving aberrant disulphide bonding [65]. Hence, it is tempting to speculate that the up-regulation of PDI is a cellular response to prevent further aggregation of abnormally disulphide bonded and misfolded TDP-43. Indeed, single nucleotide polymorphisms in the gene encoding PDI have recently been associated with risk of ALS, suggesting that PDI plays a role in protection against disease [66]. These findings align with previous results showing that enzymative inactivation of PDI in ALS patient spinal cords likely contributes to disease development [27], suggesting multiple mechanisms for the involvement of PDI in ALS pathogenesis. However, further experiments are necessary to determine the mode of PDI interaction with ALS-associated proteins, including TDP-43, and the effect of PDI on TDP-43 misfolding. It should be noted that while typically regarded as being ER-resident, PDI is also present within the cell in a variety of additional locations, including in the cytoplasm and in the extracellular space [67]. PDI is redistributed from the ER to ER-derived cytoplasmic structures in a manner regulated by the reticulon protein family, and this redistribution is protective in mutant SOD1 mice [35] and modulates PDI function [68]. The location of C-terminal TDP-43 inclusions in close association with the ER, Golgi and ERGIC (Fig. 3) also suggests disturbance in the function of these organelles, and indeed our results, and those of others [69], suggest an increased cytoplasmic presence of PDI following ER stress induction. Further investigation of changes in the Golgi and ER components in animal models displaying TDP-43 inclusions and in ALS and FTLD patient tissues is warranted.

In conclusion, this study demonstrates that ER stress can be triggered by over-expression of wildtype TDP-43 and to an even greater extent ALS-linked mutant TDP-43, and that ER stress modulates TDP-43 sub-cellular distribution and SG formation, reminiscent of disease features. These findings imply that TDP-43 mutation carriers may be more susceptible to ER stress induction than other ALS patients. However, further experiments will be required, possibly comparing the effects of wildytpe and mutant TDP-43 in *in vivo* models, in order to conclusively identify differences in dysfunction of wildtype versus mutant TDP-43 with respect to ER stress in disease. Indeed, our results also indicate that ER stress is able to drive cytoplasmic relocation of wildtype TDP-43, suggesting that upstream cellular stress responses could drive TDP-43 pathology in sporadic ALS patients. It remains important to determine the upstream triggers of TDP-43-dysfunction in ALS, and to determine if modulation of ER stress responses can be used as a target for disease modifying therapies in TDP-43 proteinopathies.

## Supporting Information

Video S1 **Association of 218–414 TDP-43-mCherry inclusions with the Golgi apparatus.** Neuro2a cells were transfected with the 218–414 TDP-43-mCherry construct, and treated with 10 µM MG132 for 24 h starting at 24 h post-transfection. Cells were processed for immunocytochemistry against GM130 (green) and serial z-stack images were taken at 0.50 µm intervals using confocal microscopy and post-processed using Olympus Fluoview v2.0 software. mCherry fluorescence is shown in red and nuclei (Hoechst dye) are shown in blue. Scale bar represents 10 µm.
(MOV)
